# The Role of Wnt Signaling in the Development of Alzheimer's Disease: A Potential Therapeutic Target?

**DOI:** 10.1155/2014/301575

**Published:** 2014-05-04

**Authors:** Wenbin Wan, Shijin Xia, Bill Kalionis, Lumei Liu, Yaming Li

**Affiliations:** ^1^Geriatrics Department of Traditional Chinese Medicine, Huadong Hospital, Fudan University, Shanghai 200040, China; ^2^Shanghai Institute of Geriatrics, Huadong Hospital, Fudan University, Shanghai 200040, China; ^3^Department of Perinatal Medicine Pregnancy Research Centre and University of Melbourne Department of Obstetrics and Gynaecology, Royal Women's Hospital, Parkville, VIC 3052, Australia

## Abstract

Accumulating evidence supports a key role for Wnt signaling in the development of the central nervous system (CNS) during embryonic development and in the regulation of the structure and function of the adult brain. Alzheimer's disease (AD) is the most common form of senile dementia, which is characterized by **β**-amyloid (A**β**) deposition in specific brain regions. However, the molecular mechanism underlying AD pathology remains elusive. Dysfunctional Wnt signaling is associated with several diseases such as epilepsy, cancer, metabolic disease, and AD. Increasing evidence suggests that downregulation of Wnt signaling, induced by A**β**, is associated with disease progression of AD. More importantly, persistent activation of Wnt signaling through Wnt ligands, or inhibition of negative regulators of Wnt signaling, such as Dickkopf-1 (DKK-1) and glycogen synthase kinase-3**β** (GSK-3**β**) that are hyperactive in the disease state, is able to protect against A**β** toxicity and ameliorate cognitive performance in AD. Together, these data suggest that Wnt signaling might be a potential therapeutic target of AD. Here, we review recent studies related to the progression of AD where Wnt signaling might be relevant and participate in the development of the disease. Then, we focus on the potential relevance of manipulating the Wnt signaling pathway for the treatment of AD.

## 1. Introduction 

Wnt ligands interact with their receptor and/or coreceptor in the cytomembrane and subsequently activate intracellularly the signaling pathway known as the Wnt signaling pathway. In vertebrates, Wnt signaling starts during development and acts by programing and regulating cell proliferation, differentiation, translocation, polarization, and fate decisions, during both embryonic development and tissue homoeostasis in the mature individual [1, 2]. Dysfunctional Wnt signaling is associated with several human diseases, such as cancer, metabolic disease, osteoporosis, schizophrenia, autism, mood disorders, epilepsy, and Alzheimer's disease (AD) [1–4]. Owing to the importance of Wnt signaling in a wide range of biological fields, a better understanding of the precise mechanism of Wnt signaling might provide fundamental insights regarding its role in disease development and may provide novel targets for therapeutic applications.

Mounting evidence indicates that Wnt signaling plays an essential role in the development of central nervous system (CNS). These roles include early anterior-posterior axis formation and neural growth and development in vertebrates and the regulation of the structure and function of the adult nervous system [5–8]. AD is the most common form of senile dementia and is characterized by senile plaque (SP) formation, which are composed of extracellularly deposited *β*-amyloid (A*β*) and also neurofibrillary tangles (NFT) that contain the intracellular aggregated hyperphosphorylated microtubule-associated protein (MAP): tau. These plaques lead to lost neural function and cognitive impairment [9–11]. The pathogenesis of AD has been intensively studied in the last decade [9–11]. However, the mechanisms underlying the neuron defects and synapse damage in AD remain elusive [9–14]. Wnt signaling plays an essential role in regulating the formation and function of neuronal circuits [6]. Furthermore, dysfunctional Wnt signaling induced by A*β* has been detected in AD and is associated with neuron degeneration and synapse impairment [15, 16]. These data suggest a strong relationship between AD and Wnt signaling pathway impairment. In addition, glycogen synthase kinase-3*β* (GSK-3*β*) is one of the most important serine/threonine (Ser/Thr) kinases that not only phosphorylates MAP tau, leading to NFT formation in AD, but is also an essential negative regulator of Wnt signaling [5, 11, 17]. Extensive evidence suggests that A*β* pathology precedes hyperphosphorylated tau pathology [18]. However, the regulatory mechanism whereby A*β* induces hyperphosphorylated tau is still unclear. Recent studies both* in vivo* and* in vitro* show that Wnt signaling may bridge the gap between the two pathological products, where GSK-3*β* is an important mediator in the production of AD pathological products [19–22]. More importantly, persistent activation of Wnt signaling through Wnt ligands, or inhibition of its negative regulators, such as Dickkopf-1 (DKK-1) and GSK-3*β* that are hyperactive in disease state, is able to protect against A*β* toxicity and ameliorate cognitive performance in AD [19–25]. These observations suggest Wnt signaling might be a novel and promising target for AD therapy. Here, we review recent studies on the progression of AD, where Wnt signaling may participate in the development of AD. Finally, we focus on the potential of modulating Wnt signaling as a potential treatment for AD.

## 2. Role of Wnt Signaling in the CNS 

### 2.1. Wnt Signaling Pathway

**Figure 1:**
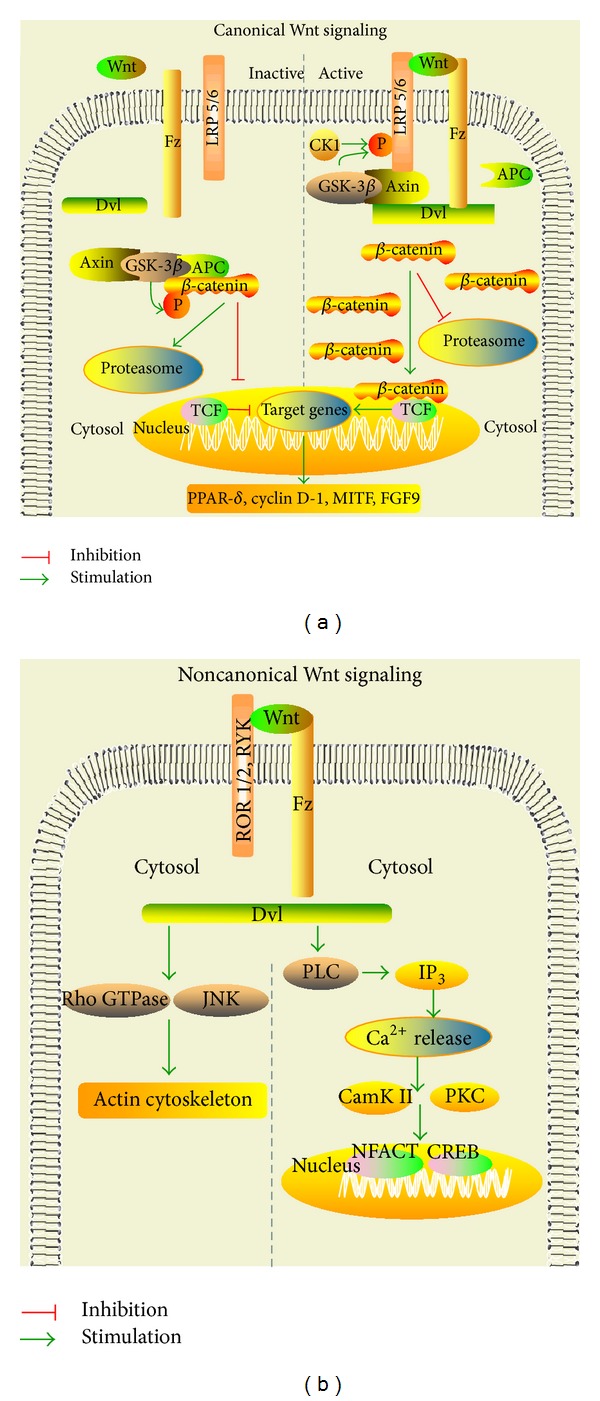
The Wnt signaling pathway. Wnt binds to the classical receptor Frizzled (Fz) and activates the down-stream signaling pathways. (a) Wnt binds to Fz and its coreceptor and eventually with casein kinase-1 (CK-1) participation, activating the scaffold protein Dishevelled (Dvl). Subsequently this induces the disassembly of the “destruction complex” and leads to the accumulation and the stabilization of *β*-catenin in the cytosol and its translocation into the nucleus, which promotes target gene expression, such as* PPAR-*δ**,* cyclin D-1*,* GLUT-1*,* Claudin-3, *and* Claudin-5*. (b) In the planar cell polarity (PCP) pathway, Wnt ligand binds to Fz, which in turn stimulates Dvl with its coreceptor, followed by activation of Rho/Rac small GTPase and c-Jun-N-terminal kinase (JNK), which leads to changes in actin and microtubule reorganization. In the Ca^2+^ pathway, the intracellular level of Ca^2+^is increased by Wnt-receptor interaction via phospholipase-C (PLC), which in turn causes Ca^2+^ release followed by Ca^2+^/Calmodulin-dependent protein kinase II (CamK II) and protein kinase C (PKC) activation, which in turn activate the nuclear translocation of transcription factor nuclear factor of activated T cells (NFACT) and cAMP response element-binding protein-1 (CREB) and consequently activate gene transcription.

Wnt signaling is classified as canonical or noncanonical depending on the downstream involvement of the *β*-catenin pathway. Canonical Wnt signaling (Wnt/*β*-catenin pathway) is dependent on the *β*-catenin pathway, whereas noncanonical Wnt signaling involves *β*-catenin independent pathways, which comprise different types of Wnt ligands (i.e., Wnt-4, Wnt-5, Wnt-11) and receptors (see Figure 1) [26]. Wnt ligands are highly conserved molecules among different species [27] that are secreted as glycosylated/lipid-modified proteins and act via autocrine and paracrine mechanisms [28]. *β*-catenin acts as the core factor; its intracellular content and phosphorylation status determine the downstream cascade in canonical Wnt signaling [26]. Wnt (i.e., Wnt-1, Wnt-3a, Wnt-7a) binds to aminoterminal cysteine-rich domain (CRD) of the seven transmembrane-receptor Frizzled (Fz) and its coreceptor (LRP 5/6); subsequent binding with casein kinase-1 (CK-1) leads to activation of the scaffold protein Dishevelled (Dvl). Activation of Dvl in turn induces the disassembly of the “destruction complex” comprising adenomatous polyposis coli (APC), axin, diversin, and the Ser/Thr kinase GSK-3*β* [3, 5]. GSK-3*β* is inhibited in the presence of Wnt protein, resulting in disassembly of the “destruction complex”, which leads to the accumulation and the stabilization of *β*-catenin in the cytosol and its translocation into the nucleus (see Figure 1(a)) [29].

In the absence of Wnt, *β*-catenin is rapidly targeted for ubiquitin-dependent degradation by the “destruction complex” following phosphorylation by both CK-1 and GSK-3*β*. Phosphorylated *β*-catenin is recognized by E3 ligase *β*-transducin repeat-containing protein (*β*-TrCP), which ubiquitinates phosphorylated *β*-catenin, targeting it for proteasome degradation and consequently results in a low cellular level of *β*-catenin [30–32]. In the nucleus, *β*-catenin is associated with the transcription factor T cell factor/lymphoid enhancing factor (TCF/LEF) and regulates gene expression that the Wnt signaling pathway targets. These targets, including* peroxisome proliferator-activated receptor *δ** (*PPAR*-**δ**),* cyclin D-1*,* MITF, *and* FGF9* [1, 5, 20, 29, 33], have been implicated in the development of limbs, neural tube, forebrain, midbrain, and cerebellum, and in the maintenance of neurotransmission and synaptic plasticity [1, 5, 34, 35].

There are at least two *β*-catenin independent pathways, the planar cell polarity pathway (Wnt/PCP pathway, also known as Wnt/JNK pathway) and the calcium pathway (Wnt/Ca^2+^ pathway, see Figure 1(b)) [26]. The signaling is transduced either via small G-proteins such as Rho/Rac (Wnt/PCP pathway) that subsequently control planar cell polarity via actin cytoskeletal remodeling or through regulation of the intracellular Ca^2+^ level (Wnt/Ca^2+^ pathway), which in turn affects diverse biological processes [1, 26, 29, 36]. In the Wnt/PCP pathway, the Wnt ligand binds to its receptor Fz and activates the scaffolding protein Dvl, followed by activation of Rho/Rac small GTPase and c-Jun-N-terminal kinase (JNK), which in turn lead to changes in both actin and microtubule reorganization [29, 37, 38]. The Wnt/PCP pathway is responsible for asymmetric distribution of cytoskeleton and cell polarization via cell planar polarity and cytoskeletal reorganization, respectively [39]. With regard to Ca^2+^ signaling, the intracellular level of Ca^2+^ is increased by Wnt ligand-receptor interaction via phospholipase-C (PLC), which causes an increase in intracellular Ca^2+^ release, and decreases cyclic guanosine monophosphate (cGMP) [40, 41]. Ca^2+^ sensitive kinases such as Ca^2+^/Calmodulin-dependent protein kinase II (CamK II) and protein kinase C (PKC) [5] are subsequently activated [40, 41]. These molecular events activate the nuclear translocation of transcription factor nuclear factor of activated T cells (NFACT) and transcription factors such as cAMP response element-binding protein (CREB) [36, 40, 41].

Interestingly, the same ligand can act through different Wnt signaling pathways depending on the specific receptor and the cellular context [5, 42]. In addition to Fz, other proteins have also been described as alternative coreceptors for the Wnt signaling pathway. These alternate coreceptors include low-density lipoprotein receptor-related protein 5/6 (LRP 5/6) and the single-pass transmembrane receptors tyrosine kinases (RTKs) Ror1, RYK, and Ror2 [3, 42, 43]. In canonical Wnt signaling, both Fz and LRP 5/6 recruit Dvl, which is then phosphorylated by CK-1 and consequently there is oligomerization in the membrane, forming a platform for the allocation of the scaffold protein Axin and GSK-3*β* [44, 45]. Moreover, LRP 5/6 phosphorylation inhibits the components of “destruction complex” such as GSK-3*β* and APC [46].

### 2.2. Wnt Signaling in CNS

Many lines of evidence support a role for Wnt signaling in neuronal synapse formation and remodeling by promoting the recruitment of presynaptic and postsynaptic components. A role for Wnt signaling has also been proposed in neuronal function maintenance and in the prevention of synaptic failure in neurodegenerative diseases, such as AD [5–7, 24, 36, 42, 47, 48]. Addition of Wnt-7a to cultured cerebellar granule cells promotes axonal spreading and branching [49]. Moreover Wnt-7a increases the frequency of miniature excitatory postsynaptic current (mEPSCs), which reflects the dynamics of neurotransmitter release induced by Wnt-7a treatment [50]. Wnt-3a increases and guides axonal branching and growth cone remodeling in spinal sensory neurons [48]. Studies show that both Wnt-7a and Wnt-3a increase growth cone size and axon branching [48]. Wnt-5a increases the amplitude of field excitatory postsynaptic potentials (fEPSP) and enhances synaptic NMDA-receptor currents, which facilitates the induction of excitatory long-term potentiation (LTP) [51, 52]. Moreover, Wnt-5a is required for nerve growth factor- (NGF-) dependent axonal growth and branching [53]. Other investigations show Wnt-5a can function as a key NGF downstream effector in the development of sympathetic neurons through local PKC activation [53]. Wnt-5a knockout neurons show faults in NGF-dependent axonal development [53]. Wnt-7a expression is detected in granule cells during postnatal days 12–22 when synapses are formed in mouse cerebellum [54]. Wnt-7a promotes synapsin I clustering and impels growth cone enlargement, both of which are key to synapse formation [54]. Furthermore, Wnt-7a-null murine neurons show deficits in synapsin I clustering and less complicated mossy fiber axonal rosettes [54]. Moreover, Wnt proteins are extensively involved in dendrite formation, maintenance, and function [37, 55]. For example, Wnt-2 stimulates dendritic complexity in cultured hippocampal neurons [55]. Finally, Wnt-7b promotes dendritic arborization by increasing dendritic length and the formation of complex branches in the hippocampus during dendritogenesis [37].

In addition to Wnt ligands, the Wnt signaling pathway comprises many downstream effectors, including Dvl and GSK-3*β* [56]. The scaffold protein Dvl is involved in both canonical and noncanonical signaling [1]. Dvl consists of three well-established domains, the N-terminal DIX (Dvl and Axin) domain, the central PDZ (postsynaptic density-95, discs large, and Zonula Occludens-1) domain, and the C-terminal DEP (Dvl, Eg-10 and pleckstrin) domain [1]. Dvl promotes neurite outgrowth and induces neuroblastoma 2A cell (N2A cells) differentiation. Neuronal remodeling in N2A cells is dependent on a Dvl N-terminal DVL domain (DIX) and DIX plays an essential role in N2A cells differentiation [56]. Further studies show Dvl increases microtubule stability via GSK-3*β* inhibition and MAP-1B restoration, which contributes to axonal microtubules stabilization and protects it from nocodazole depolymerization [37, 57]. *β*-Catenin is postulated to be a critical factor in dendritic morphology [58]. Dendritic arborization is enhanced after increasing intracellular levels of *β*-catenin [58]. In contrast, sequestering endogenous *β*-catenin results in decreased dendritic complexity [58]. GSK-3*β*, originally identified as one of the rate limiting enzymes of glycogen synthase (GS), is a key modulator of the Wnt/*β*-catenin signaling contributing to “destruction complex” formation and *β*-catenin degradation [2, 32].

## 3. Wnt Signaling in AD

A*β* is naturally released into interstitial fluid (ISF) of the brain in an activity-dependent manner under physiological conditions, which facilitates synapse function and neuronal activity [59]. In AD, however, the A*β* peptide is considered to be an important characteristic pathological hallmark that contributes to the disease development [10, 13]. Redundant A*β* aggregates and deposits in the brain parenchyma resulting in CNS damage, including neuronal death and synaptic damage in particular regions of the brain that are associated with the clinical symptoms of AD. These symptoms include progressive deterioration of the individual cognitive function and amnesia [10, 59]. A*β* also damages memory, alters hippocampal synaptic plasticity, blocks induction of LTP, and increases long-term depression (LTD) [60]. Furthermore, a reduction of fEPSP and mEPSCs is observed in the presence of A*β* [61]. These data indicate that cognitive decline in AD might be due to a reduction in synaptic transmission that is induced by A*β* [60, 61]. Although this is an area of intense investigation, the determining factors responsible for the development of AD remain elusive [10, 13]. However, evidence is accumulating that dysfunctional Wnt signaling activities, such as a reduction in the levels of *β*-catenin, constitutively active GSK-3*β*, and increasing expression of Wnt signaling inhibitor, DKK-1, are associated with AD pathology [2, 5, 8, 20–22, 62, 63].

### 3.1. Wnt Signaling Dysfunction in AD

The determining factors that trigger AD are still unclear but there are some promising candidates, which are common factors involved in several other neuronal diseases and are also essential components of the Wnt signaling machinery [36]. One such candidate is GSK-3*β*, the expression and protein activity of which is increased in the hippocampus of AD individuals [36, 64]. A significant reduction in *β*-catenin translocation to the nucleus, which is indicative of impaired Wnt signaling functions, has been detected in transgenic murine models expressing familial AD mutations [36]. Constitutively active GSK-3*β* contributes to aberrant tau phosphorylation and NFT formation, as well as a low level of *β*-catenin in the hippocampus of AD patients [36]. Overexpression of GSK-3*β* prevents the induction of LTP and reduces spatial learning, which links the characteristic memory defects in AD to an increase in GSK-3*β* [65, 66]. Recent* in vivo* work shows that A*β* interacts with the receptor for advanced glycation end-products (RAGE), which is a crucial factor that is overexpressed in the AD brain [67]. RAGE exacerbates the neuronal toxicity of A*β* and subsequently activates GSK-3*β*, which can lead to the cascade of pathologies associated with AD, whereas simultaneous inhibition of GSK-3*β* reverses the neuronal damage aggravated by A*β* [67]. Coexpression of A*β*_42_ with tau_wt_ in a* Drosophila* model of AD increases tau phosphorylation and exacerbates all the tau-mediated phenotypes. Treatment of tau_wt_/A*β*_42_ flies with lithium, a reversible inhibitor of GSK-3*β*, ameliorates the exacerbating effect of A*β*_42_, suggesting that GSK-3*β* is involved in the mechanism by which A*β*_42_ and tau_wt_ interact to cause neuronal dysfunction [18]. Interestingly, activated GSK-3*β* also stimulates the amyloidogenic processing of amyloid precursor proteins (APP) cleavage by *β*- and *γ*-secretases [68, 69]. These data collectively suggest that GSK-3*β* mediates A*β* toxicity via a positive feedback loop, not only via activation induced by A*β* but also facilitating A*β* production. Indeed, lithium protects rat neurons from A*β* toxicity [2], and activation of Wnt signaling resulting from GSK-3*β* inhibition in cultured hippocampal neurons, and leads to neuroprotection in an* in vivo* transgenic model of AD [19, 21, 70]. Thus, the suggested mechanism of GSK-3*β*-mediated regulation of A*β* toxicity may play an important role in AD pathology. Therefore, inhibition of GSK-3*β*, which leads to activation of Wnt signaling, may be a promising drug target for AD.

DKK-1 is a secreted glycoprotein and a negative modulator of Wnt signaling that binds LRP and blocks the interaction of Wnt/Fz and is associated with AD pathology [22, 23, 65, 71]. A significant increase of DKK-1 expression is found in postmortem AD brains, and brains from transgenic mouse models for AD, where DKK-1 colocalizes with hyperphosphorylated tau and GSK-3*β* staining [22, 59, 71]. DKK-1 immunoreactivity is detected in neurons surrounding A*β* deposition [22]. Synapse loss mediated by A*β* contributes to cognitive impairment, but little is known about the mechanism by which A*β* triggers the loss of synapses [72]. DKK-1 expression, together with the loss of synaptic sites via acute exposure to A*β* oligomers, was determined in a recent work [23]. Importantly, silencing of DKK-1, or neutralising the DKK-1 protein, protects against A*β*-induced apoptosis and tau phosphorylation and blocks the deleterious effects of A*β* on synapses [22, 23, 71]. These data indicate that induction of DKK-1 contributes to the pathological cascade triggered by A*β* and that DKK-1 has a critical involvement in the process of tau phosphorylation that involves GSK-3*β*. This supports the notion that DKK-1 is a key mediator of AD and that DKK-1 is a potential therapeutic target.

Allele 4 of apolipoprotein *ε* (APO-*ε*4), a plasma cholesterol transport molecule, is associated with increased risk of developing the sporadic form of AD by lowering the age of onset [73–75]. Consistent with this suggestion, APO-*ε*4 causes inhibition of the canonical Wnt signaling pathway in PC-12 cells upon stimulation with Wnt-7a, as determined by luciferase activities and nuclear *β*-catenin levels [73]. Interestingly, a common variant of the LRP-6 co-receptor (Val-1062), which has reduced *β*-catenin signaling* in vitro*, was shown to interact with APO-*ε*4 carrier status to form a risk haplotype for AD [75]. Additionally, APO-*ε*4 is considered to indirectly induce ectopic DKK-1 expression and subsequent Wnt inhibition via enhanced A*β* toxicity; however the intrinsic molecular mechanism of APO-*ε*4 action related to this effect is still unclear [76].

### 3.2. Attenuation of A*β* Neurotoxicity via Wnt Signaling Activation

Activation of Wnt signaling leads to neuroprotective effects in AD [21, 70]. Electrophysiological analysis of Schaffer collateral-CA-1 glutamatergic synaptic transmission in hippocampal slices indicates that Wnt-5a attenuates the deregulations of fEPSP and EPSCs induced by A*β*, which shows the neuroprotective properties of Wnt signaling activation are induced by Wnt-5a [61]. Coperfusion of hippocampal slices with Wnt-5a and A*β* prevents the synaptic depression of EPSCs, as well as the reduction of postsynaptic scaffold protein (PSD-95) clusters induced by A*β* in neuronal cultures [61]. Together, these results indicate that synaptic damage induced by A*β* toxicity in hippocampal neurons is prevented by Wnt pathway activation [61]. Mitochondrial dysfunction is present in numerous neurodegenerative diseases, including AD [15]. A recent study shows that activation with Wnt-5a results in the modulation of mitochondrial dynamics, preventing the changes induced by A*β* in mitochondrial fission-fusion dynamics, and modulating the Bcl-2 increase induced by A*β* [15]. In rat hippocampal neurons, direct activation of Wnt signaling by its endogenous Wnt-3a ligand prevents the toxic effects induced by A*β*. A*β* toxic effects on hippocampal neurons, such as impairment of neuronal cell survival, an increase in GSK-3*β* and tau phosphorylation, a decrease in cytoplasmic *β*-catenin, and a decrease in the expression of Wnt target gene* engrailed*-*1*, are overcome by Wnt-3a [21]. However, the role played by Fz-1, via Wnt signaling, has not been studied. Fz-1 mediates the activation of the Wnt/*β*-catenin signaling by Wnt-3a [77]. The protective effect of Wnt-3a against the toxicity of A*β* is modulated by Fz-1 expression levels in both PC-12 cells and hippocampal neurons [77]. Overexpression of Fz-1 significantly increases cell survival induced by Wnt-3a and diminishes Capase-3 activation, in the presence of A*β*, while silencing Fz-1 reverses the Wnt-3a protective effect [77].

## 4. Wnt Signaling as a New Therapeutic Target in AD

Currently, no cure exists for AD and the exact molecular mechanism leading to its onset is not fully understood. Accumulated evidence indicates that dysfunctional Wnt signaling, induced by A*β* in AD, contributes to disease progression [2, 21, 36, 61, 76]. Furthermore, persistent activation of Wnt signaling, via Wnt ligands or by inhibiting negative regulators [e.g., DKK-1, GSK-3*β*, and soluble Frizzled related protein (sFRP)], is indeed able to overcome the toxic effects induced by A*β* and ameliorate cognitive performance in AD [2, 21, 25, 61, 76, 78–84]. These data suggest the Wnt signaling pathway may be a potential therapeutic target for AD.

Wnt ligands, including Wnt-3a, -7a, are able to protect neurons against A*β* toxicity and facilitate fEPSP in hippocampal neurons [21, 61, 85]. Wnt-3a overcomes toxic effects induced by A*β*, such as impaired neuronal survival, an increase in GSK-3*β*, and a reduction of *β*-catenin [21]. Another study showed Wnt-3a protein partially protects PC-12 cells from the toxic effects of A*β*, with a 6–15% increase in cell viability. Wnt-3a treatment of A*β*-treated PC-12 cells increased *β*-catenin protein expression by 52% compared with the control [78].

DKK-1 is a secreted glycoprotein, which is induced by A*β* and shows increased expression in AD. DKK-1 binds to LRP and prevents its interaction with Wnt ligands [79]. DKK-1 silencing not only attenuates the reduction in the inactive phosphorylated form of GSK-3*β* but also reduces apoptosis in neurons challenged with A*β* [71]. Moreover, DKK-1-neutralizing antibodies suppress synapse loss in mouse brain slices induced by A*β*, which indicates that blockading DKK-1 could be beneficial for the maintenance of synapses in AD [23]. DKK-1 and Wnt ligands bind to two distinct recognition sites on the LRP 5/6 coreceptors, indicating that antagonists of DKK-1 might interfere with the interaction between DKK-1 and LRP 5/6 without affecting Wnt binding to the receptors [63]. A specific small molecule inhibitor, NCI8642, can efficiently displace DKK-1 from LRP-6 and block DKK-1 inhibitory activity on canonical Wnt signaling [86]. Additionally, the high-bone-mass mutation (G171V) of the Wnt coreceptor LRP-5 has been reported to cause an increase in Wnt activity in osteoblasts by reducing the number of targets for paracrine DKK-1 to antagonize without affecting the activity of autocrine Wnt [87], yet its role in neurons remains to be determined.

Inhibition of GSK-3*β* results in neuroprotective effects in both hippocampal cultured neurons and in an* in vivo* transgenic model of AD [36]. GSK-3*β* inhibitors stimulate neuronal differentiation and the mood stabilizer, lithium, which acts through the Wnt/*β*-catenin signaling pathway, enhances proliferation of adult hippocampal progenitors* in vitro,* and induces them to become neurons at therapeutically relevant concentrations [80, 81]. Chronic treatment with lithium results in decreased neurogenesis in the subgranular zone of the hippocampus in a transgenic murine model of AD compared with nontransgenic mice [80]. Lithium significantly stimulates the proliferation and neuron fate specification of newborn cells and fully counteracts the transgene-induced impairments to cognitive functions [80]. Curcumin has been shown to activate Wnt signaling in a similar manner to GSK-3*β* inhibition in APPswe transfected SHSY5Y cells [82]. The expression of GSK-3*β* mRNA and protein significantly decreased in transfected cells treated with Curcumin in a dose and time-dependent manner [82]. And an increase in performance of the protein expression of GSK-3*β*-Ser9 is detected as well. Meanwhile, expression of *β*-catenin and Cyclin-D1 mRNA and protein is increased [82]. Immunofluorescent staining shows the translocation of *β*-catenin into the nucleus increases gradually with the increased dosage of Curcumin [82]. AF267B, a specific agonist of M-1 muscarinic receptor, was also determined to activate Wnt signaling, through GSK-3*β* inhibition [83, 84]. Chronic AF267B administration in the 3×Tg-AD model was shown to rescue cognitive deficits in a spatial task and reduce A*β* and tau pathologies in the hippocampus due to GSK-3*β* inhibition [83]. Thus, M-1 muscarinic receptor and Wnt signaling interaction result in neuroprotection against A*β* toxicity via GSK-3*β* inhibition [83, 84].

Nonsteroidal anti-inflammatory drugs (NSAIDs), *α*7-nicotinic acetylcholine receptors (*α*7-nAChRs), inhibitor of acetylcholinesterase (AChE), and peroxisome proliferator activated receptors (PPARs) are also involved in the activation of Wnt signaling pathway and protect against A*β* toxicity [88, 89]. A novel bifunctional compound, Ibuprofen-Octyl-Pyridostigmine (IBU-PO) which combines an NSAID (Ibuprofen) and a cholinesterase (ChE) inhibitor (Octyl-Pyridostigmine), has been reported to inhibit GSK-3*β* and stabilize *β*-catenin, reverting the silencing of the Wnt signaling caused by A*β* toxicity and GSK-3*β* overexpression [90]. In addition, IBU-PO enhances, in a dose dependence manner, nonamyloid APP cleavage by increasing secreted APP and decreasing endogenous A*β*_1-40_ in rat hippocampal neurons [90]. A reversible and selective inhibitor of AChE, Huperzine A (HupA), was shown to activate Wnt signaling via GSK-3*β* inhibition and stabilize the level of *β*-catenin and reduce amyloidosis in the AD brain [89]. The PPAR*γ* agonist, troglitazone, also prevents changes in Wnt signaling triggered by A*β* [91]. In the same study, activation of neuronal PPAR*γ* prevented *β*-catenin destabilization induced by A*β* and induced translocation of cytoplasmic *β*-catenin to the nucleus [91], resulting in protection of hippocampal neuron morphology in cells exposed to A*β* [91]. Nicotine, an unselective *α*7-nAChR agonist, prevents memory deficits and synaptic impairment in AD [92]. Additionally, new findings reveal that there is cross-talk between *α*7-nAChR and Wnt/*β*-catenin signaling since nicotine stabilizes *β*-catenin and prevents A*β*-induced loss of *β*-catenin through the *α*7-nAChR [92]. Furthermore, activation of canonical Wnt signaling induces *α*7-nAChR expression [92]. Taken together, these data indicate that activation of the Wnt signaling pathway may as well be therapeutic target for potential AD treatments.

## 5. Conclusions and Future Directions

AD is an irreversible neurodegeneration disease characterized by fibrillar deposits of A*β* in subcortical brain regions [93]. A*β* is considered to be the main factor in AD that causes neuronal dysfunction, neurodegeneration, and cognition impairment, which eventually leads to death from complete bran failure [13]. The molecular mechanisms underlying pathological changes in AD remain to be elucidated. As reviewed above, Wnt signaling is essential for neuronal development and maintenance of the nervous system [5–7, 36, 42, 47]. Dysfunctional Wnt signaling induced by A*β* toxicity in AD, characterized by reduced *β*-catenin levels, constitutively active GSK-3*β*, and increased expression of the Wnt signaling inhibitor DKK-1, suggests that sustained dysfunctional Wnt signaling may be a key event that contributes to the pathology of AD [22, 65]. Persistent activation of Wnt signaling protects neurons from A*β* toxicity, which suggests the Wnt pathway is a promising therapeutic target for the treatment of AD [2, 21, 25, 61, 76, 78–84]. Therefore, further studies of Wnt signaling, and particularly dysfunctional Wnt signaling in AD, are needed to fully understand the biological mechanisms that underlie the pathological changes in AD.

## References

[1] Kim W, Kim M, Jho EH (2013). Wnt/beta-catenin signalling: from plasma membrane to nucleus. *The Biochemical Journal*.

[2] Inestrosa NC, Montecinos-Oliva C, Fuenzalida M (2012). Wnt signaling: role in Alzheimer disease and schizophrenia. *Journal of Neuroimmune Pharmacology*.

[3] MacDonald BT, Tamai K, He X (2009). Wnt/beta-catenin signaling: components, mechanisms, and diseases. *Developmental Cell*.

[4] Clevers H (2006). Colon cancer—understanding how NSAIDs work. *The New England Journal of Medicine*.

[5] Rosso SB, Inestrosa NC (2013). WNT signaling in neuronal maturation and synaptogenesis. *Frontiers in Cellular Neuroscience*.

[6] Ciani L, Salinas PC (2005). WNTs in the vertebrate nervous system: from patterning to neuronal connectivity. *Nature Reviews Neuroscience*.

[7] Toledo EM, Colombres M, Inestrosa NC (2008). Wnt signaling in neuroprotection and stem cell differentiation. *Progress in Neurobiology*.

[8] Inestrosa NC, Arenas E (2010). Emerging roles of Wnts in the adult nervous system. *Nature Reviews Neuroscience*.

[9] Mayeux R, Stern Y (2012). Epidemiology of Alzheimer disease. *Cold Spring Harbor Perspectives in Medicine*.

[10] Mattson MP (2004). Pathways towards and away from Alzheimer’s disease. *Nature*.

[11] Buée L, Troquier L, Burnouf S (2010). From tau phosphorylation to tau aggregation: what about neuronal death?. *Biochemical Society Transactions*.

[12] Hartz AMS, Bauer B, Soldner ELB (2012). Amyloid-*β* contributes to blood-brain barrier leakage in transgenic human amyloid precursor protein mice and in humans with cerebral amyloid angiopathy. *Stroke*.

[13] Wan W, Chen H, Li Y (2014). The potential mechanisms of Abeta-receptor for advanced glycation end-products interaction disrupting tight junctions of the blood-brain barrier in Alzheimer's disease. *The International Journal of Neuroscience*.

[14] Kook SY, Hong HS, Moon M, Ha CM, Chang S, Mook-Jung I (2012). Abeta_1-42_-RAGE interaction disrupts tight junctions of the blood-brain barrier via Ca_2+_-calcineurin signaling. *The Journal of Neuroscience*.

[15] Silva-Alvarez C, Arrazola MS, Godoy JA, Ordenes D, Inestrosa NC (2013). Canonical Wnt signaling protects hippocampal neurons from Abeta oligomers: role of non-canonical Wnt-5a/Ca_2+_ in mitochondrial dynamics. *Frontiers in Cellular Neuroscience*.

[16] Thies W, Bleiler L (2011). 2011 Alzheimer’s disease facts and figures. *Alzheimer’s and Dementia*.

[17] Mendoza J, Sekiya M, Taniguchi T, Iijima KM, Wang R, Ando K (2013). Global analysis of phosphorylation of tau by the checkpoint kinases Chk1 and Chk2 in vitro. *Journal of Proteome Research*.

[18] Folwell J, Cowan CM, Ubhi KK (2010). A*β* exacerbates the neuronal dysfunction caused by human tau expression in a Drosophila model of Alzheimer’s disease. *Experimental Neurology*.

[19] Vargas JY, Fuenzalida M, Inestrosa NC (2014). In vivo activation of Wnt signaling pathway enhances cognitive function of adult mice and reverses cognitive deficits in an Alzheimer's disease model. *The Journal of Neuroscience*.

[20] Clevers H, Nusse R (2012). Wnt/beta-catenin signaling and disease. *Cell*.

[21] Alvarez AR, Godoy JA, Mullendorff K, Olivares GH, Bronfman M, Inestrosa NC (2004). Wnt-3a overcomes *β*-amyloid toxicity in rat hippocampal neurons. *Experimental Cell Research*.

[22] Rosi MC, Luccarini I, Grossi C (2010). Increased dickkopf-1 expression in transgenic mouse models of neurodegenerative disease. *Journal of Neurochemistry*.

[23] Purro SA, Dickins EM, Salinas PC (2012). The secreted Wnt antagonist dickkopf-1 is required for amyloid *β*-mediated synaptic loss. *The Journal of Neuroscience*.

[24] Maguschak KA, Ressler KJ (2012). A role for WNT/beta-catenin signaling in the neural mechanisms of behavior. *Journal of Neuroimmune Pharmacology*.

[25] Shruster A, Eldar-Finkelman H, Melamed E, Offen D (2011). Wnt signaling pathway overcomes the disruption of neuronal differentiation of neural progenitor cells induced by oligomeric amyloid *β*-peptide. *Journal of Neurochemistry*.

[26] Angers S, Moon RT (2009). Proximal events in Wnt signal transduction. *Nature Reviews Molecular Cell Biology*.

[27] van Amerongen R, Nusse R (2009). Towards an integrated view of Wnt signaling in development. *Development*.

[28] Port F, Basler K (2010). Wnt trafficking: new insights into Wnt maturation, secretion and spreading. *Traffic*.

[29] Gordon MD, Nusse R (2006). Wnt signaling: multiple pathways, multiple receptors, and multiple transcription factors. *The Journal of Biological Chemistry*.

[30] Salic A, Lee E, Mayer L, Kirschner MW (2000). Control of *β*-catenin stability: reconstitution of the cytoplasmic steps of the Wnt pathway in Xenopus egg extracts. *Molecular Cell*.

[31] Price MA (2006). CKI, there’s more than one: casein kinase I family members in Wnt and Hedgehog signaling. *Genes and Development*.

[32] Wu D, Pan W (2010). GSK3: a multifaceted kinase in Wnt signaling. *Trends in Biochemical Sciences*.

[33] Freese JL, Pino D, Pleasure SJ (2010). Wnt signaling in development and disease. *Neurobiology of Disease*.

[34] Liebner S, Corada M, Bangsow T (2008). Wnt/*β*-catenin signaling controls development of the blood—brain barrier. *Journal of Cell Biology*.

[35] Scott EL, Brann DW (2013). Estrogen regulation of Dkk1 and Wnt/beta-catenin signaling in neurodegenerative disease. *Brain Research*.

[36] Oliva CA, Vargas JY, Inestrosa NC (2013). Wnt signaling: role in LTP, neural networks and memory. *Ageing Research Reviews*.

[37] Rosso SB, Sussman D, Wynshaw-Boris A, Salinas PC (2005). Wnt signaling through dishevelled, Rac and JNK regulates dendritic development. *Nature Neuroscience*.

[38] Yamanaka H, Moriguchi T, Masuyama N (2002). JNK functions in the non-canonical Wnt pathway to regulate convergent extension movements in vertebrates. *EMBO Reports*.

[39] Huelsken J, Held W (2009). Canonical Wnt signalling plays essential roles. *European Journal of Immunology*.

[40] Kohn AD, Moon RT (2005). Wnt and calcium signaling: *β*-catenin-independent pathways. *Cell Calcium*.

[41] Montcouquiol M, Crenshaw EB, Kelley MW (2006). Noncanonical Wnt signaling and neural polarity. *Annual Review of Neuroscience*.

[42] Farías GG, Godoy JA, Cerpa W, Varela-Nallar L, Inestrosa NC (2010). Wnt signaling modulates pre- and postsynaptic maturation: therapeutic considerations. *Developmental Dynamics*.

[43] van Amerongen R, Mikels A, Nusse R (2008). Alternative wnt signaling is initiated by distinct receptors. *Science Signaling*.

[44] Gao C, Chen Y (2010). Dishevelled: the hub of Wnt signaling. *Cellular Signalling*.

[45] Bilić J, Huang Y, Davidson G (2007). Wnt induces LRP6 signalosomes and promotes dishevelled-dependent LRP6 phosphorylation. *Science*.

[46] Logan CY, Nusse R (2004). The Wnt signaling pathway in development and disease. *Annual Review of Cell and Developmental Biology*.

[47] Salinas PC, Zou Y (2008). Wnt signaling in neural circuit assembly. *Annual Review of Neuroscience*.

[48] Purro SA, Ciani L, Hoyos-Flight M, Stamatakou E, Siomou E, Salinas PC (2008). Wnt regulates axon behavior through changes in microtubule growth directionality: A new role for adenomatous polyposis coli. *The Journal of Neuroscience*.

[49] Lucas FR, Salinas PC (1997). WNT-7a induces axonal remodeling and increases synapsin I levels in cerebellar neurons. *Developmental Biology*.

[50] Cerpa W, Godoy JA, Alfaro I (2008). Wnt-7a modulates the synaptic vesicle cycle and synaptic transmission in hippocampal neurons. *The Journal of Biological Chemistry*.

[51] Cerpa W, Gambrill A, Inestrosa NC, Barria A (2011). Regulation of NMDA-receptor synaptic transmission by Wnt signaling. *The Journal of Neuroscience*.

[52] Varela-Nallar L, Alfaro IE, Serrano FG, Parodi J, Inestrosa NC (2010). Wingless-type family member 5A (Wnt-5a) stimulates synaptic differentiation and function of glutamatergic synapses. *Proceedings of the National Academy of Sciences of the United States of America*.

[53] Bodmer D, Levine-Wilkinson S, Richmond A, Hirsh S, Kuruvilla R (2009). Wnt5a mediates nerve growth factor-dependent axonal branching and growth in developing sympathetic neurons. *The Journal of Neuroscience*.

[54] Hall AC, Lucas FR, Salinas PC (2000). Axonal remodeling and synaptic differentiation in the cerebellum is regulated by WNT-7a signaling. *Cell*.

[55] Wayman GA, Impey S, Marks D (2006). Activity-dependent dendritic arborization mediated by CaM-kinase I activation and enhanced CREB-dependent transcription of Wnt-2. *Neuron*.

[56] Fan S, Ramirez SH, Garcia TM, Dewhurst S (2004). Dishevelled promotes neurite outgrowth in neuronal differentiating neuroblastoma 2A cells, via a DIX-domain dependent pathway. *Molecular Brain Research*.

[57] Ciani L, Krylova O, Smalley MJ, Dale TC, Salinas PC (2004). A divergent canonical WNT-signaling pathway regulates microtubule dynamics: dishevelled signals locally to stabilize microtubules. *Journal of Cell Biology*.

[58] Yu X, Malenka RC (2003). *β*-catenin is critical for dendritic morphogenesis. *Nature Neuroscience*.

[59] Palop JJ, Mucke L (2010). Amyloid-*Β*-induced neuronal dysfunction in Alzheimer’s disease: from synapses toward neural networks. *Nature Neuroscience*.

[60] Shankar GM, Li S, Mehta TH (2008). Amyloid-*β* protein dimers isolated directly from Alzheimer’s brains impair synaptic plasticity and memory. *Nature Medicine*.

[61] Cerpa W, Farías GG, Godoy JA, Fuenzalida M, Bonansco C, Inestrosa NC (2010). Wnt-5a occludes A*β* oligomer-induced depression of glutamatergic transmission in hippocampal neurons. *Molecular Neurodegeneration*.

[62] Kania KD, Wijesuriya HC, Hladky SB, Barrand MA (2011). Beta amyloid effects on expression of multidrug efflux transporters in brain endothelial cells. *Brain Research*.

[63] Caraci F, Busceti C, Biagioni F (2008). The Wnt antagonist, Dickkopf-1, as a target for the treatment of neurodegenerative disorders. *Neurochemical Research*.

[64] Hooper C, Killick R, Lovestone S (2008). The GSK3 hypothesis of Alzheimer’s disease. *Journal of Neurochemistry*.

[65] Killick R, Ribe EM, Al-Shawi R (2014). Clusterin regulates beta-amyloid toxicity via Dickkopf-1-driven induction of the Wnt-PCP-JNK pathway. *Molecular Psychiatry*.

[66] Hooper C, Markevich V, Plattner F (2007). Glycogen synthase kinase-3 inhibition is integral to long-term potentiation. *European Journal of Neuroscience*.

[67] Li XH, Du LL, Cheng XS (2013). Glycation exacerbates the neuronal toxicity of beta-amyloid. *Cell Death & Disease*.

[68] Phiel CJ, Wilson CA, Lee VM-Y, Klein PS (2003). GSK-3*α* regulates production of Alzheimer’s disease amyloid-*β* peptides. *Nature*.

[69] Inestrosa NC, Varela-Nallar L (2014). Wnt signaling in the nervous system and in Alzheimer's disease. *Journal of Molecular Cell Biology*.

[70] Quintanilla RA, Muñoz FJ, Metcalfe MJ (2005). Trolox and 17*β*-estradiol protect against amyloid *β*-peptide neurotoxicity by a mechanism that involves modulation of the Wnt signaling pathway. *The Journal of Biological Chemistry*.

[71] Caricasole A, Copani A, Caraci F (2004). Induction of Dickkopf-1, a negative modulator of the Wnt pathway, is associated with neuronal degeneration in Alzheimer’s brain. *The Journal of Neuroscience*.

[72] Lacor PN, Buniel MC, Furlow PW (2007). A*β* oligomer-induced aberrations in synapse composition, shape, and density provide a molecular basis for loss of connectivity in Alzheimer’s disease. *The Journal of Neuroscience*.

[73] Caruso A, Motolese M, Iacovelli L (2006). Inhibition of the canonical Wnt signaling pathway by apolipoprotein E4 in PC12 cells. *Journal of Neurochemistry*.

[74] Donahue JE, Johanson CE (2008). Apolipoprotein E, amyloid-*β*, and blood-brain barrier permeability in Alzheimer disease. *Journal of Neuropathology and Experimental Neurology*.

[75] de Ferrari GV, Papassotiropoulos A, Biechele T (2007). Common genetic variation within the low-density lipoprotein receptor-related protein 6 and late-onset Alzheimer's disease. *Proceedings of the National Academy of Sciences of the United States of America*.

[76] Boonen RACM, van Tijn P, Zivkovic D (2009). Wnt signaling in Alzheimer’s disease: up or down, that is the question. *Ageing Research Reviews*.

[77] Chacón MA, Varela-Nallar L, Inestrosa NC (2008). Frizzled-1 is involved in the neuroprotective effect of Wnt3a against A*β* oligomers. *Journal of Cellular Physiology*.

[78] Kawamoto EM, Gleichmann M, Yshii LM, de Sá Lima L, Mattson MP, Scavone C (2012). Effect of activation of canonical Wnt signaling by the Wnt-3a protein on the susceptibility of PC12 cells to oxidative and apoptotic insults. *Brazilian Journal of Medical and Biological Research*.

[79] Mao B, Wu W, Li Y (2001). LDL-receptor-related protein 6 is a receptor for Dickkopf proteins. *Nature*.

[80] Fiorentini A, Rosi MC, Grossi C, Luccarini I, Casamenti F (2010). Lithium improves hippocampal neurogenesis, neuropathology and cognitive functions in APP mice. *PLoS ONE*.

[81] Wexler EM, Geschwind DH, Palmer TD (2008). Lithium regulates adult hippocampal progenitor development through canonical Wnt pathway activation. *Molecular Psychiatry*.

[82] Zhang X, Yin W, Shi X, Li Y (2011). Curcumin activates Wnt/*β*-catenin signaling pathway through inhibiting the activity of GSK-3*β* in APPswe transfected SY5Y cells. *European Journal of Pharmaceutical Sciences*.

[83] Farías GG, Godoy JA, Hernández F, Avila J, Fisher A, Inestrosa NC (2004). M1 muscarinic receptor activation protects neurons from *β*-amyloid toxicity. A role for Wnt signaling pathway. *Neurobiology of Disease*.

[84] Caccamo A, Oddo S, Billings LM (2006). M1 receptors play a central role in modulating AD-like pathology in transgenic mice. *Neuron*.

[85] Dinamarca MC, Sagal JP, Quintanilla RA, Godoy JA, Arrzola MS, Inestrosa NC (2010). Amyloid-*β*-acetylcholinesterase complexes potentiate neurodegenerative changes induced by the A peptide. Implications for the pathogenesis of Alzheimer’s disease. *Molecular Neurodegeneration*.

[86] Iozzi S, Remelli R, Lelli B (2012). Functional characterization of a small-molecule inhibitor of the DKK1-LRP6 interaction. *ISRN Molecular Biology*.

[87] Zhang Y, Wang Y, Li X (2004). The LRP5 high-bone-mass G171V mutation disrupts LRP5 interaction with Mesd. *Molecular and Cellular Biology*.

[88] Inestrosa NC, Varela-Nallar L, Grabowski CP, Colombres M (2007). Synaptotoxicity in Alzheimer’s disease: the Wnt signaling pathway as a molecular target. *IUBMB Life*.

[89] Wang C, Zheng W, Wang T (2011). Huperzine a activates Wnt/*Β*-catenin signaling and enhances the nonamyloidogenic pathway in an Alzheimer transgenic mouse model. *Neuropsychopharmacology*.

[90] Farías GG, Godoy JA, Vázquez MC (2005). The anti-inflammatory and cholinesterase inhibitor bifunctional compound IBU-PO protects from *β*-amyloid neurotoxicity by acting on Wnt signaling components. *Neurobiology of Disease*.

[91] Inestrosa NC, Godoy JA, Quintanilla RA, Koenig CS, Bronfman M (2005). Peroxisome proliferator-activated receptor *γ* is expressed in hippocampal neurons and its activation prevents *β*-amyloid neurodegeneration: role of Wnt signaling. *Experimental Cell Research*.

[92] Inestrosa NC, Godoy JA, Vargas JY (2013). Nicotine prevents synaptic impairment induced by amyloid-beta oligomers through alpha7-nicotinic acetylcholine receptor activation. *Neuromolecular Medicine*.

[93] Dinamarca MC, Colombres M, Cerpa W, Bonansco C, Inestrosa NC (2008). *β*-amyloid oligomers affect the structure and function of the postsynaptic region: role of the Wnt signaling pathway. *Neurodegenerative Diseases*.

